# EEG-BIDS, an extension to the brain imaging data structure for electroencephalography

**DOI:** 10.1038/s41597-019-0104-8

**Published:** 2019-06-25

**Authors:** Cyril R. Pernet, Stefan Appelhoff, Krzysztof J. Gorgolewski, Guillaume Flandin, Christophe Phillips, Arnaud Delorme, Robert Oostenveld

**Affiliations:** 1 Centre for Clinical Brain Sciences, University of Edinburgh, Edinburgh, Scotland; 2 Center for Adaptive Rationality, Max Planck Institute for Human Development, Berlin, Germany; 3 Department of Psychology, Stanford University, Stanford, California USA; 4 Wellcome Centre for Human Neuroimaging, London, United Kingdom; 5 GIGA Institute, University of Liège, Liège, Belgium; 6 Swart Center for Computational Neuroscience, University of California San Diego, San Diego, California USA; 7 CerCo, CNRS/Université Paul Sabatier, Toulouse, France; 8 Donders Institute for Brain, Cognition and Behaviour, Radboud University, Nijmegen, The Netherlands; 9 NatMEG, Karolinska Institutet, Stockholm, Sweden

**Keywords:** Research management, Data publication and archiving

## Abstract

The Brain Imaging Data Structure (BIDS) project is a rapidly evolving effort in the human brain imaging research community to create standards allowing researchers to readily organize and share study data within and between laboratories. Here we present an extension to BIDS for electroencephalography (EEG) data, EEG-BIDS, along with tools and references to a series of public EEG datasets organized using this new standard.

EEG was first applied in humans nearly a century ago^1^. It records the electric potential fluctuations at the scalp, primarily from locally synchronous post-synaptic activity in the apical dendrites of pyramidal cells in the cortex. Widely used in both clinical and non-clinical settings, EEG is becoming increasingly important in cognitive neuroscience, with statistics from scientific reports showing that interest in EEG has been growing faster since the early 2000s. This can be attributed to interest in brain–computer interfaces and more sophisticated dynamics measures along with more accurate biophysical models for reconstructing sources. EEG is more versatile than other imaging modalities because (i) it is lightweight and requires relatively low-cost equipment, (ii) it can be used in many different environments (e.g., while sitting in a lab chair, driving, walking, playing a video game, sleeping, interacting with others in social situations, etc.), (iii) it can be used either alone or in conjunction with other imaging modalities, (iv) its task design constraints are less restrictive than metabolic (PET) or hemodynamic (fMRI) imaging methods, and (v) it captures neural activity with millisecond precision, making it possible to record cortical dynamics at the speed of perception, thought and action. Because of this versatility, the field of applications for EEG is broad. In turn, the commercial market for EEG systems is much larger than that of other imaging techniques (e.g., PET, MRI, MEG), resulting in a multitude of equipment manufacturers (more than 10 major manufacturers in neuroscience) building different hardware systems, usually with their own software and proprietary data formats. Manufacturers have little financial incentive to cooperate and provide compatible formats. This resulting diversity of formats is an impediment to reusing data as well as to building large-scale EEG databases.

The Brain Imaging Data Structure, originally proposed for magnetic resonance imaging data (MRI), is a human brain research community standard used for organizing and sharing brain imaging study data within and between laboratories for many (ultimately all) imaging modalities^2^. BIDS primarily addresses the heterogeneity of data organization by following the FAIR principles^3^ of findability, accessibility, interoperability, and reusability. BIDS addresses *findability* and *reusability* by providing rich metadata in dedicated sidecar files and *interoperability* by using existing standard data formats. *Accessibility* is not directly addressed by BIDS, but by repositories that build on BIDS, such as OpenNeuro (https://openneuro.org). By stipulating how to structure data using naming conventions and dedicated metadata files to store dictionaries (.json) and data (.tsv), BIDS fosters interoperability and reuse of acquired datasets. Because BIDS data are structured, BIDS also addresses issues of reproducibility by allowing the creation of fully automated data analysis workflows.

Here we report on the extension of BIDS to EEG data. EEG-BIDS builds upon the MEG-BIDS extension^4^ and is presented concurrently with the iEEG-BIDS extension covering human intracranial electrophysiology^5^. In this document we only highlight the main features of EEG-BIDS. The full documentation of the EEG-BIDS extension can be found via the link to the specification on the general BIDS website (https://bids.neuroimaging.io/).

## EEG-BIDS Summary

**Fig. 1:**
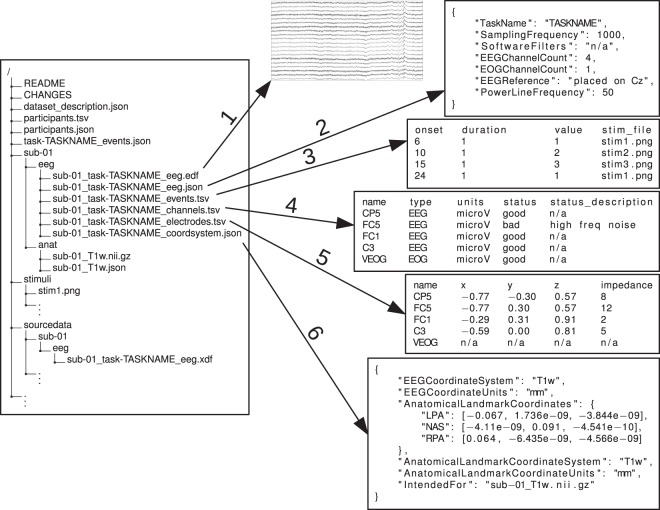
Exemplary EEG-BIDS dataset with previews of EEG files. The left side of the figure shows a standard BIDS directory tree with the root containing files describing the dataset in general (“README”, “dataset_description.json”), a file describing the participants (“participants.tsv”), and as several JSON files (“participants.json”, “task-TASKNAME_events.json”), which contain the description necessary to understand the contents of the TSV files. Note that JSON files at high levels get inherited by lower levels unless overridden (the Inheritance Principle). Next to the files at the root, there is a stimuli and a sourcedata directory that can be used to save the respective study data. Most important are the subject directories named “sub- <sub-label> ” for each study participant. Nested in the subject directories are all recorded data split over modalities (eeg and anat, for the EEG and structural MRI data respectively). The right side of the figure shows the contents of the eeg modality directory, including the raw EEG data (1) and associated metadata (2). An events.tsv file (3) specifies all events that were recorded during the session and can reference presented stimuli with the stimuli directory of the dataset (see stim_file column). A “channels.tsv” file (4) provides further information about the raw EEG data and can contain information not present in the raw EEG data file such as filter settings and channel status (good/bad). Finally, an “electrodes.tsv” file (5) and an accompanying “coordsystem.json” file (6) provide electrode locations and specify which coordinate framework to use to interpret the electrode locations (for example with respect to a T1 weighted MRI scan).

The extension of BIDS to EEG data closely follows the general BIDS specification (see Figure 1). Each subject’s data corresponds to a directory of raw data containing subdirectories for each session and data modality. This is accompanied by a “dataset_description.json” file containing generic information about the dataset and in the case of the EEG modality, a metadata file with the suffix “eeg.json”. The “eeg.json” file exhaustively specifies among other metadata details of the experimental task and the EEG recording system. Optional directories include the “sourcedata” directory, which can be used to supply original non-formatted data. Furthermore, a “stimuli” directory and a “code” directory can be present to allow data conversion and preprocessing to be reproduced, as indicated in the original specification^2^. Within each subject directory, the “eeg” subdirectory contains the EEG and metadata. For instance, for a single session study, “sub-XX” would have subdirectory “eeg” which contains EEG files using the naming pattern “sub-XX_task-YY_eeg. <extension>” corresponding to acquisitions of EEG data. In addition, “sub-XX_task-YY_channels.tsv” must be specified describing the parameters of the data acquisition and two extra files, “sub-XX_task-YY_electrodes.tsv” and “sub-XX_task-YY_coordsystem.json”, should be specified if the positions of the electrodes are known (see below).

As in the MRI specification of BIDS, “sub-XX_task-YY_events.tsv” files should be present, encoding all of the parameters of the experimental design (onset of events, trial type, duration, responses, etc.). While such information is often present as one or several binary “trigger” channels in the EEG recordings, the representation of events is rarely explicit in the original data (e.g., a numeric code is used to indicate the onset of a given picture presented in a given experimental condition) hence the necessity of these “events.tsv” files. Since the initial specification of BIDS for MRI in 2016, the Hierarchical Event Descriptor (HED) system for precise annotation of events has been integrated, which is particularly useful for electrophysiological data in which dynamics of brain activity are associated with multiple experimental events^6^.

## Specific EEG-BIDS Considerations

The process of converging on a list of suitable data formats for EEG-BIDS was governed by three major requirements: A suitable data format should (i) address the needs of a large portion of the global EEG community, (ii) be interoperable according to the FAIR principles^3^, and (iii) meet the technical requirements of neuroscientific workflows, such as saving numerical data with high precision.

As a solution to this challenge, the EEG-BIDS specification incorporates only two recommended “official” data formats: The European Data Format (EDF), which is an ongoing international effort to provide a common data format for electrophysiological recordings that began in 1992^7^, and the BrainVision Core Data Format, developed by Brain Products GmbH. While the BrainVision Core Data Format was designed by Brain Products GmbH for its proprietary EEG recording equipment and analysis software, it is based on the Microsoft Windows INI file and has a concise documentation. Both of these formats follow the three requirements for suitable data formats for EEG-BIDS: (i) A recent survey indicates that they are widely used in the community (https://bids.berkeley.edu/news/bids-megeegieeg-data-format-survey), (ii) they have open access documentation and an open source implementation for both reading and writing in at least two programming languages that are widely used in the field (in this case, Python and MATLAB, among others), and (iii) they have high numerical precision (EDF:16 bits, BrainVision Core Data Format:32 bits). To accommodate a larger scientific audience and facilitate adoption, the EEG-BIDS standard also allows two “unofficial” commonly used data formats: The format used by the MATLAB toolbox EEGLAB^8^ (“.set” and “.fdt” files), and the Biosemi format (“.bdf”). While not actively encouraged, these two formats are included due to their popularity and their interoperability among the major software packages. Future versions of BIDS may extend the list of “officially” supported data formats, based on the fulfillment of the above mentioned three requirements for suitable data formats. Independently of the raw data format used, critical metadata about the recording are always available in BIDS .tsv and .json files.

In order to provide an unambiguous documentation of EEG data, we are clarifying two sets of terms that are often used interchangeably: Electrodes versus channels, and fiducials versus anatomical landmarks.

We distinguish between electrodes and channels using the following definitions: (i) An EEG electrode is a contact point attached to the skin, (ii) a channel is the combination of the analog differential amplifier and analog-to-digital converter that result in a potential (voltage) difference being stored in the EEG dataset. The “reference” and “ground” electrodes should in general not be referred to as channels and only as electrodes. Some systems (e.g., Biosemi) have an active floating reference, whilst for most of the other systems, the potential at electrodes is neither amplified nor recorded. For EEG-BIDS, researchers must specify a “channels.tsv” file and may in addition specify an “electrodes.tsv” file with an accompanying “coordsystem.json” file.

Furthermore, we distinguish between fiducials and anatomical landmarks using the following definitions: (i) Fiducials are objects with a well defined location used to facilitate the localization of electrodes and co-registration with other geometric data such as the participant’s own T1 weighted magnetic resonance head image, a T1 weighted template head image, or a spherical head model. Commonly used fiducials are vitamin E pills, which are clearly visible in an anatomical MR image, or reflective spheres that are localized with an infrared optical tracking system. (ii) Anatomical landmarks on the other hand define locations on a research subject such as the nasion, which is the intersection of the frontal bone and two nasal bones of the human skull. Fiducials are typically used in conjunction with anatomical landmarks.

## Public EEG-BIDS Datasets

Several study examples (with zero-byte data files) are available in the BIDS-examples GitHub repository (https://github.com/bids-standard/bids-examples). We have also released three full datasets formatted using the EEG-BIDS standard:

- The Matching Pennies dataset^9^ is an example of a single recording session per participant. It was collected as part of a student project to replicate a brain-computer interface study of motor intention decoding.
- The Rishikesh dataset^10^ is an example of multiple recording sessions per participant. Participants were asked to meditate continuously whilst experience-sampling probe questions were presented at random intervals throughout the duration of the experiment.
- The simultaneous resting-state EEG-fMRI dataset^11^ offers resting-state data in EEG and fMRI modalities, and structural T1 weighted data and diffusion data (NODDI sequence).

## Community Tools and Software Support

As part of the BIDS project, datasets formatted to follow the EEG-BIDS standard can be validated using the “bids-validator”, a JavaScript application that runs locally as a command line version (using Node.js) or within an Internet browser (https://bids-standard.github.io/bids-validator/). With this validation tool, researchers can check their newly formatted datasets and make full use of the data structure’s strengths for instance, checking for missing data or underspecified metadata.

The BIDS starter kit (https://github.com/bids-standard/bids-starter-kit) is a collection of community-driven guides, tutorials, helper scripts, and wiki resources to help researchers get started with BIDS. The resources cover two popular programming languages (Python and MATLAB) and will be extended over time to incorporate additional guides.

We are collaborating with the developers of the most widely used EEG data analysis tools in order to help EEG practitioners convert their existing data to the EDF or BrainVision Core Data Format. Data conversion utilities from many raw EEG data formats to the EDF and BrainVision format are available in MATLAB from the FieldTrip^12^ and EEGLAB^8^ toolboxes, and in Python from the MNE-Python^13^ package. While specific software implementations are beyond the scope of the data specification, we here also want the reader to be aware of further work being undertaken by the open source community to interact with BIDS datasets. For instance, an EEGLAB^8^ “study” can now be exported as BIDS (std_tobids.m), FieldTrip^12^ can similarly export (data2bids.m) while SPM12^14^ (spm_bids.m) and MNE-Python^13^ (in form of the MNE-BIDS project https://github.com/mne-tools/mne-bids) can read any BIDS dataset. Ultimately, reading/writing BIDS dataset will be fully automatic, as it is the case for MRI.

## Data Analysis Pipelines and Beyond Sharing Raw Data

EEG’s long history, versatility and variety of applications makes it a data- and method-rich technique. Recently, the OHBM Committee on Best Practice in Data Analysis and Sharing (COBIDAS) released a guideline for good practice and reproducibility in EEG^15^. According to the guideline, even the simplest processing pipeline already contains eight separate steps, making it clear that while EEG-BIDS helps with data sharing, it will remain non-trivial to develop automated processing pipelines of neurophysiology data such as already available for MRI data^16^. On its own, EEG-BIDS is the first necessary step toward achieving validated and reproducible data analysis through standardizing the complete documentation of a dataset.

This article describes the new EEG extension for BIDS, and has limited itself to sharing raw data using previously developed community standards. Challenges that are specific to EEG, such as support for data formats, are still under active debate, and some additional formats will likely be incorporated once the technical issues are addressed and FAIR standards are achieved. The development of BIDS for EEG derivatives is also already underway (BIDS Extension Proposal 21): This will also make sharing of processed data possible, thereby fostering re-analyses, meta-analyses, and new analyses without the burden of data preparation.
